# Racialized Perceptions of Vegetarianism: Stereotypical Associations That Undermine Inclusion in Eating Behaviors

**DOI:** 10.1177/01461672221099392

**Published:** 2022-07-07

**Authors:** Daniel L. Rosenfeld, Tiffany N. Brannon, A. Janet Tomiyama

**Affiliations:** 1 University of California, Los Angeles, USA

**Keywords:** race, stereotype, eating behavior, vegetarianism, belonging

## Abstract

Shifting societal eating patterns toward a vegetarian diet offers promise for improving public health and environmental sustainability. Yet concerns exist about racial disparities in inclusion, as some sentiments suggest that vegetarianism is stereotypically associated with Whiteness. Through four studies (total *N* = 3,234), we investigated associations U.S. adults hold between race and vegetarianism, along with implications for behavior change and belongingness among Black individuals. Participants, across racial backgrounds, strongly associated vegetarianism with Whiteness, both explicitly and implicitly. A race prime led Black participants to report lower interest in becoming a vegetarian, whereas a prime of race-vegetarianism associations decreased Black participants’ feelings of belongingness in the vegetarian community. Exposure to racially inclusive messaging about vegetarianism, meanwhile, increased belongingness among Black participants. These findings provide the first quantitative insights into racial stereotypes about vegetarianism and pose future directions for theory, research, and practice at the intersections of race and eating behavior.

Think of a prototypical vegetarian. Imagine what type of person would order tofu at a restaurant, pick up meatless nuggets for a quick meal at Kentucky Fried Chicken, or grill a veggie burger for a barbecue. What race has your mind ascribed to this fictitious individual?

Based on stereotypes conveyed in popular discourse (e.g., Davis, 2017; Williams, 2019), the prototypical vegetarian in the United States is White. The potential for vegetarianism to be racialized as White, importantly, may make it seem noninclusive to people of color—a concern poised to become increasingly pressing as vegetarian alternatives hold growing promise as solutions for major social issues. Specifically, societal shifts toward a vegetarian diet are touted as critical for optimizing public health and environmental sustainability (Willett et al., 2019). High rates of meat consumption contribute toward costly chronic disease, biodiversity loss, inefficient water use, and greenhouse gas emissions (Clark et al., 2019; Willett et al., 2019). For example, widespread adoption of vegetarian diets would reduce expected greenhouse gas emissions from food production by 55% (Tilman & Clark, 2014).

Despite the weight of these considerations, no research to our knowledge has tested quantitatively whether people indeed hold racial stereotypes about vegetarianism, be they explicit or implicit. Understanding these stereotypes is vital for conceptualizing psychosocial determinants of eating behavior and for ultimately promoting an inclusive social climate, given that eating is a highly social phenomenon (Delormier et al., 2009). Thus, in the current research, we asked the following: Is vegetarianism truly perceived to be a “White” behavior, and could this racialized perception undermine how inclusive it seems to become a vegetarian?

## The Social Construction of Vegetarianism

Evaluating the social construction of vegetarianism in light of its historical roots may illuminate its racialization today. Vegetarianism as a dietary pattern is far from a recent development: It traces back over 2,000 years to Greek philosophers in ancient times (Spencer, 1993). Long before modern cultural conceptions of vegetarianism, vegetarian eating had been espoused by prominent intellectual, artistic, and social justice figures from myriad racial and cultural backgrounds—including Leonardo da Vinci, Benjamin Franklin, Albert Einstein, Mahatma Gandhi, Coretta Scott King, Rosa Parks, and Cesar Chavez (Couceiro et al., 2007; Von Alt, 2015). Cross-culturally, moreover, predominantly vegetarian diets have traditionally been cornerstones in many of the longest-living societies around the world, including Abkhazia in the Caucasus south of Russia, Vilcabamba in the Andes of South America, and Hunza in Central Asia (Robbins, 2007). Meatless diets transcend racial and ethnic divides and are endorsed by a variety of religions and religious denominations such as Buddhism, Hinduism, and Seventh Day Adventist Christianity (Dictionary.com, n.d.). Throughout time and place, vegetarian eating behavior has been diverse in its scope.

Modern conceptions of vegetarianism, meanwhile, are relatively young. Although people have been choosing to consume meatless diets for millennia, the word “vegetarian” was first coined in the 1830s and the word “vegan” in the 1940s (Dictionary.com, n.d.; The Vegan Society, n.d.). The introduction of these terms likely shifted the psychological landscape, assigning greater uniqueness and social significance to vegetarian dietary behaviors. To call oneself a vegetarian is to proclaim a lexicalized label that signals social identity, and expressing one’s eating pattern through such language can conjure up stronger psychological and temporally stable notions of one’s behavior (Gelman & Heyman, 1999). With the advent of a label focusing on the self as “a vegetarian,” the idea of what it meant to forgo meat likely transcended mere dietary behavior and became a psychologically essentialized trait, providing a basis for stereotype formation (Bastian & Haslam, 2006). In this sense, the *identity* of being a vegetarian is unique from the *behavior* of eating a vegetarian diet (Nezlek & Forestell, 2020; Rosenfeld & Burrow, 2017; Rosenfeld et al., 2020): To say that one “doesn’t eat meat” is to describe what one *does*; to say that one “is a vegetarian” is to proclaim who one *is*.

Indeed, self-identifying as a vegetarian entails expressing a group membership associated with distinct personality traits, beliefs, and behaviors (MacInnis & Hodson, 2017; Minson & Monin, 2012)—and, we posit, a distinct race: White. Central to this association may be perceptions of higher social class. Without food security and some degree of socioeconomic privilege, it is difficult to experience adequate choice in which foods to consume. In modern Western cultures like the United States, compared with predominantly White neighborhoods, predominantly Black neighborhoods have fewer supermarkets and more fast-food restaurants (Walker et al., 2010), which tend to offer more meat-based meals. Not everyone possesses true “choice” in food consumption. To the extent that people perceive both vegetarianism and Whiteness as signaling higher social class, such overlap in stereotype content may drive perceptions of vegetarianism with Whiteness.

Potential associations of vegetarianism in the United States with Whiteness today may be fueled by a host of factors—from those pertaining to class, to skewed media depictions (Greenebaum, 2018), or simply to common misconceptions—that paint a picture of vegetarianism as a social identity removed from the historical, culturally diverse roots of its underlying eating pattern. Nevertheless, the existence of these associations may have meaningful implications for how people of color construe the prospect of becoming a vegetarian themselves.^1^ It might have critical consequences for behavior change and sense of inclusion tied to vegetarianism.

## Implications of Racializing Vegetarianism for Behavior Change and Inclusion

Despite lay perceptions of vegetarianism as White (e.g., Davis, 2017; Williams, 2019), quantitative research on the racialized nature of vegetarianism is absent, leaving the current knowledge base limited to theoretical and qualitative states of evidence (e.g., Greenebaum, 2018; Harper, 2009). Although qualitative methodologies are valuable and can amplify the voices of communities of color in unique ways, they nevertheless preclude estimating effect sizes quantitatively. Quantitative methodologies, meanwhile, offer tools for estimating the extents to which people associate vegetarianism with various racial groups relatively and to which these associations may directly influence individuals’ attitudes toward becoming a vegetarian. The present knowledge gap may hinder efforts to promote behavior change that can mitigate health disparities and environmental degradation that can arise from overconsumption of meat (Willett et al., 2019).

Food presents a versatile marker for social identity and a common source of racial/ethnic stereotypes (Fischler, 1988). Behaviors related to eating can serve as racial/ethnic identity primes (e.g., Macrae et al., 1995; Shih et al., 2007), directly heightening awareness of one’s membership in a social group. Moreover, disparate depictions within media—for example, overrepresenting Black individuals as consumers of fast food (Gilmore & Jordan, 2012)—may perpetuate stereotypes entwining racial identifications with eating styles. However, more recent marketing campaigns for plant-based foods that feature celebrities of color including comedian Kevin Hart, rapper Snoop Dogg, and social media influencer Liza Koshy might represent a shift (Beer, 2020), potentially allowing people of color to feel a greater sense of belongingness tied to vegetarianism.

According to self-categorization theory, individuals categorize themselves into social groups at varying levels of abstraction, perceiving their self-attributes and/or actions in terms of shared social identities (Turner et al., 1987). Race and vegetarianism afford two bases for self-categorization, and an individual’s identification as a person of color may influence their attitudes toward identifying as a vegetarian. Racialized associations of vegetarianism may foster social identity acceptance threat among people of color in fostering a perceived conflict between a preexisting group membership (one’s race) and a prospective group membership (being a vegetarian) that appear incompatible (Branscombe et al., 1999).

In experiencing social identity acceptance threat, a person of color may feel as though other vegetarians would not accept them fully as a member of the group were they to become a vegetarian themselves. We posit that associating vegetarianism with Whiteness may lead people of color to perceive vegetarian spaces as psychologically threatening, as people of color may expect members of their racial group to be disproportionately outnumbered and ostracized in such spaces. These predictions extend, yet are consistent with, research that has examined consequences of stereotypical associations between racialized and gendered associations in educational and occupational contexts. For example, exposure to a workplace with few underrepresented minority employees reduces Black individuals’ trust of and comfort toward the workplace (Purdie-Vaughns et al., 2008). Moreover, within Science, Technology, Engineering, and Mathematics (STEM) settings and mainstream workplaces, exposure to stereotypical environmental cues can undermine women’s feelings of belongingness and lower their interests in STEM (Cheryan et al., 2009, 2015; Murphy et al., 2007). We posit that in a given environment, to the extent that situational cues activate associations of vegetarianism with Whiteness, people of color may perceive engagement in vegetarianism as a source of acceptance threat and thus feel less interested in becoming a vegetarian.

We recognize that *being a member* of a social group is different from *being integrated into* that social group (Roberson, 2006); simply diversifying vegetarianism might not necessarily make it more inclusive. A person of color might be highly committed to being a vegetarian yet still feel as though they do not belong in the vegetarian community. This lack of belongingness may be especially likely to occur when individuals possess dual identities (Baysu et al., 2011), such as the duality of being both a person of color and a vegetarian. Accordingly, in addition to interest in becoming a vegetarian, we directly considered feelings of belongingness in the vegetarian community to be a key outcome in our research, as to investigate not only a behaviorally relevant outcome but also an affective outcome related to inclusion. By assessing expectations about belongingness, we captured the extent to which participants felt that they would fit in with other vegetarians if they were to become vegetarians themselves, feeling included in and accepted by the vegetarian community.

Cultivating a deeper study of race and vegetarianism can provide timely insights into real-world social change. A growing movement is afoot to delegitimize societal associations of vegetarianism with Whiteness and to make vegetarianism more inclusive, particularly to the Black community. A simple web search for “Black vegetarians,” for instance, yields pages of lists highlighting famous Black vegetarians, along with many articles that directly call out mainstream images of vegetarianism for unjustly being disproportionately White (e.g., Davis, 2017; Williams, 2019). Entire books (e.g., Harper, 2009; Ko & Ko, 2017), organizations (e.g., Black Vegans Rock; Black Vegetarian Societies of Georgia, Texas, New York, etc.), and festivals (e.g., Black VegFest) devoted to Black vegetarianism are emerging and rising to prominence, along with the rise of vegetarian soul food restaurants throughout the United States. These events illustrate the implications of dietary stereotypes in real-world settings related to eating behavior, and a deeper understanding of basic processes can illuminate their natures while fostering their successes.

In the current research, we center much of our investigation (Studies 2–4) on the link between race and vegetarianism as related to White people and Black people, in particular, for two key reasons. First, although some existing discourse on racialized perceptions of vegetarianism has drawn a distinction between White people and people of color broadly (e.g., Gossard & York, 2003; Greenebaum, 2018), distinctions have most often singled out Black people in comparison to the Whiteness norm pervading images of vegetarianism (e.g., Davis, 2007; Harper, 2009; Ko & Ko, 2017; Williams, 2019; Williams-Forson, 2006). Second, the health benefits of vegetarian eating patterns seem to carry notable potential for Black people, compared with Latinx or Asian people. Black people consume a particularly high amount of meat (U.S. Department of Agriculture, 2012), which may be one factor underlying their elevated risks for cardiovascular disease (Howard et al., 2017; Willett et al., 2019)—the leading cause of death for Black men and women (CDC, 2017a, 2017b). Identifying racial stereotypes as relevant to Black people’s attitudes toward vegetarianism can be an important step toward promoting more healthful behavior change and mitigating racial disparities in health. Findings from the current research can ultimately be used to examine the link between race and vegetarianism more broadly beyond White-Black comparisons.

## Overview of the Current Studies

We present four studies investigating stereotypes about race and vegetarianism. In Study 1, we assessed participants’ explicit associations of vegetarianism with four target racial/ethnic groups (White, Black, Latinx, and Asian). In Study 2, we examined both explicit and implicit associations of vegetarianism with White versus Black people. In Study 3, in a sample of Black participants, we manipulated the salience of participants’ own race and their race-vegetarianism associations and tested for effects of these primes on two outcomes, one of which was behaviorally relevant (interest in becoming a vegetarian) and the other affectively relevant (feelings of belongingness in the vegetarian community). In Study 4, in another sample of Black participants, we compared the effects of racially inclusive versus stereotypical messaging about vegetarianism on interest and belongingness.

In each study, we report all manipulations, measures, and exclusions employed, as well as all preregistered procedures and analyses, when applicable. Materials for all studies are available at https://osf.io/kd2ap/.

## Study 1

In Study 1, we explored explicit associations between race and vegetarianism. We sought to identify which racial groups were most to least associated with vegetarianism.

### Method

Data, analysis scripts, and codebook are available at https://osf.io/nsk5z/.

#### Participants

In determining this study’s sample size, we sought high power to detect very small differences in association strengths of vegetarianism with each racial group to minimize the chance of reporting false negatives, given the lack of existing research on this topic. A power analysis using G*Power 3.1 specifying a very small effect of *d* = 0.10 revealed that a total sample of 787 participants would provide 80% power at α = .05, two-tailed. To increase power further and account for attention-check exclusions, we recruited a total of 2,000 adults from the United States, via Amazon Mechanical Turk (MTurk), on January 25–26, 2020. After excluding 147 participants who failed an attention check in the survey, 1,853 participants (865 men, 981 women, 8 other) between the ages of 18 and 88 (*M*_age_ = 41.18, *SD* = 13.25) were retained for analyses. Of these participants, 1,441 self-identified as White, 142 as Black, 84 as Hispanic/Latinx, 125 as Asian or Pacific Islander, 10 as Native American, 48 as mixed race/ethnicity, and 3 as other race/ethnicity. This sample provided 80% power to detect very small differences of *d* = 0.07 in associations between vegetarianism and each target race at α = .05, two-tailed.

#### Materials

##### Explicit race–vegetarianism associations

Explicit race–vegetarianism associations were assessed using eight total items.

The first four items led with the following prompt, adapted from Devos and Banaji (2005): “Please bring to mind people who eat a vegetarian diet. In your mind, how ‘vegetarian’ are people who belong to the following racial groups? That is, how strongly are they identified with vegetarianism and all things vegetarian?” Following this prompt were four items on separate lines below, each presenting a scale ranging from 1 (not at all) to 5 (very much). The item on the first line read, “White people,” the second line “Black people,” the third line “Latino/a people,” and the fourth line “Asian people.”

The next four items led with the question, “When you think of a vegetarian, how much do you think of someone who is . . .?” and followed with four items on separate lines below, each presenting a scale ranging from 1 (not at all) to 5 (very much). The item on the first line read, “White,” the second line “Black,” the third line “Latino/a,” and the fourth line “Asian.”

To compute scores of explicit associations, we computed an average of the items for each race across these two prompts. Internal consistencies were high for all four race–vegetarianism associations: The two White items correlated at *r* = .78, Black items at *r* = .77, Latinx items at *r* = .76, and Asian items at *r* = .81, all *p* < .001.

#### Procedure

After consenting to take part in this study, participants completed the measure of explicit race-vegetarianism associations.

### Results and Discussion

**Figure 1.**
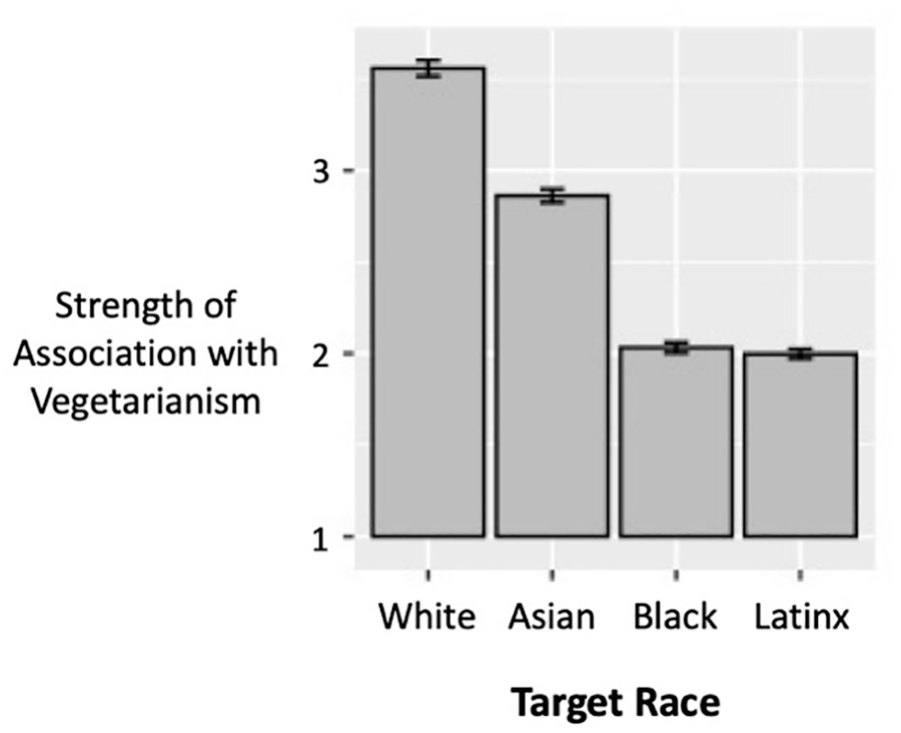
Explicit associations between vegetarianism and racial/ethnic groups (White, Asian, Black, and Latinx) in Study 1, assessed on a scale of 1–5 (error bars indicate 95% confidence intervals).

A within-subjects analysis of variance (ANOVA) indicated that participants significantly associated vegetarianism with the racial group targets to varying extents, *F*(3, 5,556) = 1,829.86, *p* < .001, η_p_^2^ = 0.50, suggesting that participants held strongly racialized perceptions. Participants associated vegetarianism most strongly with White people, followed by Asian people, and least strongly with Black and Latinx people (see Figure 1). Bonferroni-adjusted paired *t*-tests revealed significant differences in association strength of vegetarianism with White versus Black people (*d* = 1.32), White versus Latinx people (*d* = 1.31), Latinx versus Asian people (*d* = 0.89), Black versus Asian people (*d* = 0.77), and White versus Asian people (*d* = 0.56), each comparison *p* < .001. Associations of vegetarianism with Black versus Latinx people were not significantly different (*p* = .077, *d* = 0.06).

## Study 2

Results of Study 1 highlight that people hold clear stereotypes about race and vegetarianism. In Study 2, we elaborated on Study 1 by investigating not only explicit but also implicit associations between race and vegetarianism. For reasons outlined earlier (e.g., greatest relevance for real-world discussions and existing health disparities), and given the effect sizes observed in Study 1, we focused our investigation on White and Black participants, as specified in our study preregistration plan. A limitation of Study 1 was its lack of diversity, as 78% of participants were White. In Study 2, we addressed this limitation by recruiting a balanced sample with equal numbers of White and Black participants.

We hypothesized that participants would explicitly and implicitly associate vegetarianism more strongly with White people than with Black people. We also tested two secondary research questions regarding these associations. First, do participants’ explicit and/or implicit associations between vegetarianism and race differ as a function of their own race? Second, do participants’ explicit and implicit associations between race and vegetarianism differ as a function of their own dietary status (i.e., whether participants are vegetarian or not)? We set no specific predictions for these secondary hypotheses.

### Method

This study’s sample size, conditions, exclusion criteria, materials, hypotheses, and analyses were preregistered at https://osf.io/ws5yc.

Data, analysis scripts, and codebook are available at https://osf.io/k5d4m/.

#### Participants

We sought adequate power to detect small-medium effects (*d* = 0.35) between White and Black participants as well as small effects (*d* = 0.20) within each racial group. Power analyses using G*Power 3.1 revealed that a total sample of 398 participants, with equal numbers of White and Black participants, would provide at least 80% power to detect both of these effect sizes at α = .05, two-tailed. Thus, we recruited 400 U.S. adults—200 White and 200 Black—via Prolific on October 4–5, 2019. Participants were prescreened by race automatically through Prolific (without participants’ awareness) and reported their race within the survey; we continued data collection until our sample included 200 participants who self-identified as White and 200 as Black. After excluding 14 participants who failed an attention check in the survey, 386 participants (177 men, 201 women, 8 other) between the ages of 18 and 72 (*M*_age_ = 34.45, *SD* = 12.31) were retained for analyses. Of these participants, 194 were White and 192 were Black.

#### Materials

##### Explicit race–vegetarianism association

Explicit race–vegetarianism association was assessed as in Study 1, but with only White and Black as the target races. For each of the two association prompts, we subtracted each participant’s score on the Black item from their score on the White item in order to compute a score capturing the extent of association of vegetarianism as White over Black. These two associations scores correlated at *r*(384) = .78, *p* < .001. As preregistered, we averaged these two association scores to compute a final variable that operationalized explicit association of vegetarianism with White people over Black people.

##### Implicit race–vegetarianism association

Implicit race–vegetarianism association was assessed using an Implicit Association Test (IAT; Greenwald et al., 1998), created using the iatgen software (Carpenter et al., 2019). The IAT presented words to capture each race (White, Black) and diet (vegetarian, meat-eater). Words for *White* included “White” and “Caucasian.” Words for *Black* included “Black” and “African American.” Words for *vegetarian* included “vegetarian,” “vegan,” “plant-based,” “veggie,” “vegetables,” “tofu,” “salad,” and “lettuce.” Words for *meat-eater* included “meat-eater,” “omnivore,” “carnivore,” “barbecue,” “meat,” “hamburger,” “pork,” and “chicken.” We selected these words based on output from a “related words” generator (available at https://relatedwords.org), input from colleagues, and our review of scholarly and popular-press literatures. Our IAT is available for download in Qualtrics Survey Format at https://osf.io/782nh/.

#### Procedure

After consenting to take part in this study, participants completed the explicit and implicit measures of race–vegetarianism associations in a randomized order. Then, participants indicated whether or not they consider themselves to be vegetarian. Participants completed demographic questions at the end of the survey, wherein they confirmed their race.

### Results and Discussion

We evaluated the two parts of our primary hypothesis through two one-sample *t*-tests. These tests indicated that participants explicitly (*M* = 0.84, *SD* = 0.58), *t*(385) = 28.68, *p* < .001, 95% confidence interval (CI) [0.79, 0.90], *d* = 1.46, and implicitly (IAT *D*-score *M* = 0.35, *SD* = 0.40), *t*(385) = 17.13, *p* < .001, 95% CI [0.31, 0.39], *d* = 0.87, associated vegetarianism more strongly with White people than with Black people.

We evaluated our two secondary research questions through four independent *t*-tests, conducting Welch-adjusted tests when Levene’s tests indicated unequal variances between groups. First, participants’ explicit, *t*(384) = 0.24, *p* = .813, 95% CI [−0.13, 0.10], *d* = 0.02, and implicit, *t*(372.95) = 1.09, *p* = .275, 95% CI [−0.12, 0.04], *d* = 0.11, associations between race and vegetarianism did not differ as a function of their own race. Second, whereas participants’ implicit associations between race and vegetarianism did not differ as a function of their own vegetarian versus meat-eater dietary status, *t*(13.41) = 1.20, *p* = .253, 95% CI [−0.54, 0.16], *d* = 0.38, a significant difference did emerge for explicit association, *t*(384) = 2.19, *p* = .029, 95% CI [−0.49, −0.03], *d* = 0.50, such that meat-eaters (*M* = 0.85, *SD* = 0.58, *p* < .001, *d* = 1.48) associated vegetarianism more strongly with White people over Black people than did vegetarians (*M* = 0.60, *SD* = 0.45, *p* < .001, *d* = 1.33).

## Study 3

Results of Study 2 suggest that both explicit and implicit associations of vegetarianism with Whiteness are very strong, among both White and Black people. In Study 3, we investigated potential implications of these associations for behavior change and belongingness among Black individuals. Could racial stereotypes about vegetarianism undermine how inclusive it seems?

Through a 2 × 2 experiment, involving a sample of Black participants, we examined two types of primes: a race prime (heightening participants’ awareness of their race) and a race–vegetarianism association prime (heightening participants’ awareness of their associations between race and vegetarianism). By randomizing participants to race prime, association prime, race+association prime, and no-prime conditions, we tested the effects of each of these two primes independently and together on participants’ interest in becoming a vegetarian and their feelings of belongingness in the vegetarian community. We hypothesized that both the race and race–vegetarianism association primes would cause participants to report lower interest and lower belongingness (i.e., negative main effects of each prime on each outcome). We also hypothesized a negative interaction effect between the two primes on each outcome, reasoning that racial stereotypes about vegetarianism may make vegetarianism feel particularly noninclusive for Black individuals when they feel heightened awareness of their own race.

### Method

This study’s sample size, materials, conditions, exclusion criteria, hypotheses, and analyses were preregistered at https://osf.io/vzmuy.

Data, analysis scripts, and codebook are available at https://osf.io/e9z2j/.

#### Participants

In determining this study’s sample size, we accounted for small–medium main effects of each prime. A power analysis using G*Power 3.1 specifying an effect of *d* = 0.35 revealed that a total sample of 260 participants would provide 80% power at α = .05, two-tailed. To increase power further, we recruited a generous sample of 500 Black adults from the United States via Prolific on October 20, 2020. Participants were prescreened by race automatically through Prolific (without participants’ awareness) and reported their race within the survey; we continued data collection until our sample included 500 participants who self-identified as Black. After excluding 60 participants who failed an attention check in the survey and 37 participants who reported being vegetarian or vegan, 403 participants (160 men, 241 women, 2 other) between the ages of 18 and 76 (*M*_age_ = 31.12, *SD* = 10.80) were retained for analyses. This sample provided 80% power to detect small-medium main effects of *d* = 0.28 at α = .05, two-sided.

#### Materials and procedure

After consenting to take part in this study, participants were randomly assigned to complete one of the four study conditions: race prime, association prime, both primes, or control (no primes). In the race prime condition, participants completed the race prime before completing outcome measures (interest and belongingness scales). In the association prime condition, participants completed the association prime before completing outcome measures. In both primes’ condition, participants completed both the race prime and the association prime (in a randomized order) before completing outcome measures. In the control condition, participants completed outcome measures at the start of the survey, in the absence of any primes. At the end of the survey, participants completed demographic questions.

##### Race prime

Race was primed through three steps. First, participants indicated their race. Second, participants read the following prompt: “Please take a moment to reflect on what you think it means to be a Black person. Reflect on what role race plays in your life. When you are ready, please proceed with the survey.” Third, participants reflected on their racial identity by completing a four-item scale of Black racial identity centrality adapted from Sellers et al. (1997). An example scale item read, “Being Black is an important reflection of who I am.” Responses ranged from 1 (strongly disagree) to 7 (strongly agree). On average, participants for whom race was primed reported very high levels of racial identity centrality (*M* = 5.85, *SD* = 1.36).

##### Race–vegetarianism association prime

Participants’ associations between race and vegetarianism were primed via completion of explicit association items and the IAT as described in Study 2. As in Studies 1 and 2, participants for whom associations were primed did indeed associate vegetarianism very strongly with White people more than with Black people, both explicitly (*d* = 1.36) and implicitly (*d* = 1.12).

##### Interest in becoming a vegetarian

Interest was assessed by a four-item scale (α = .93), an example item reading, “I can imagine myself becoming a vegetarian someday.” Responses ranged from 1 (strongly disagree) to 7 (strongly agree).

##### Feelings of belongingness in the vegetarian community

Belongingness was assessed using an adapted eight-item (α = .93) version of Malone et al.’s (2012) General Belongingness Scale. The scale began with a prompt that read, “If I were to become a vegetarian. . .” and followed with eight items, each presenting a response scale ranging from 1 (*strongly disagree*) to 7 (*strongly agree*). An example item read, “I would feel included by other vegetarians.”

### Results and Discussion

Interest and belongingness were correlated at a small–medium magnitude of *r*(398) = .22, *p* < .001.

**Figure 2.**
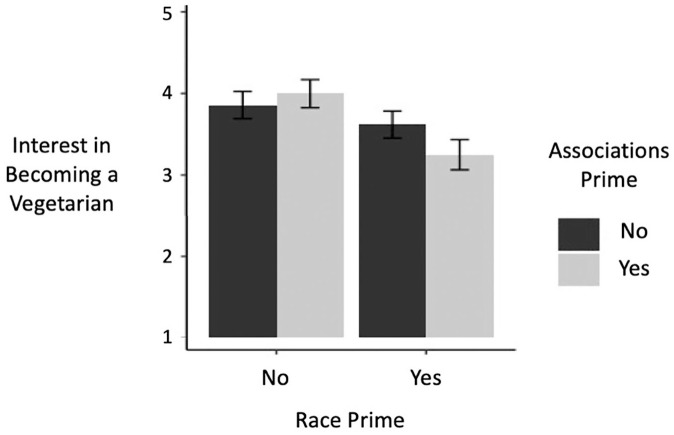
Effects of priming race and race–vegetarianism associations on interest in becoming a vegetarian in a sample of Black participants in Study 3 (error bars indicate standard errors; interest was assessed on a scale of 1–7).

A two-way ANOVA revealed a significant main effect of race prime on interest in becoming a vegetarian, *F*(1, 399) = 7.93, *p* = .005, η_p_^2^ = 0.02, such that participants for whom race was primed (*M* = 3.45, *SD* = 1.78) reported lower interest than did participants for whom race was not primed (*M* = 3.93, *SD* = 1.69), *d* = 0.28 (see Figure 2). The main effect of race–vegetarianism association prime (i.e., completion of IAT + explicit items), *F*(1, 399) = 0.49, *p* = .483, η_p_^2^ = 0.00, and the interaction effect between the two primes, *F*(1, 399) = 2.05, *p* = .153, η_p_^2^ = 0.01, were not significant.

**Figure 3.**
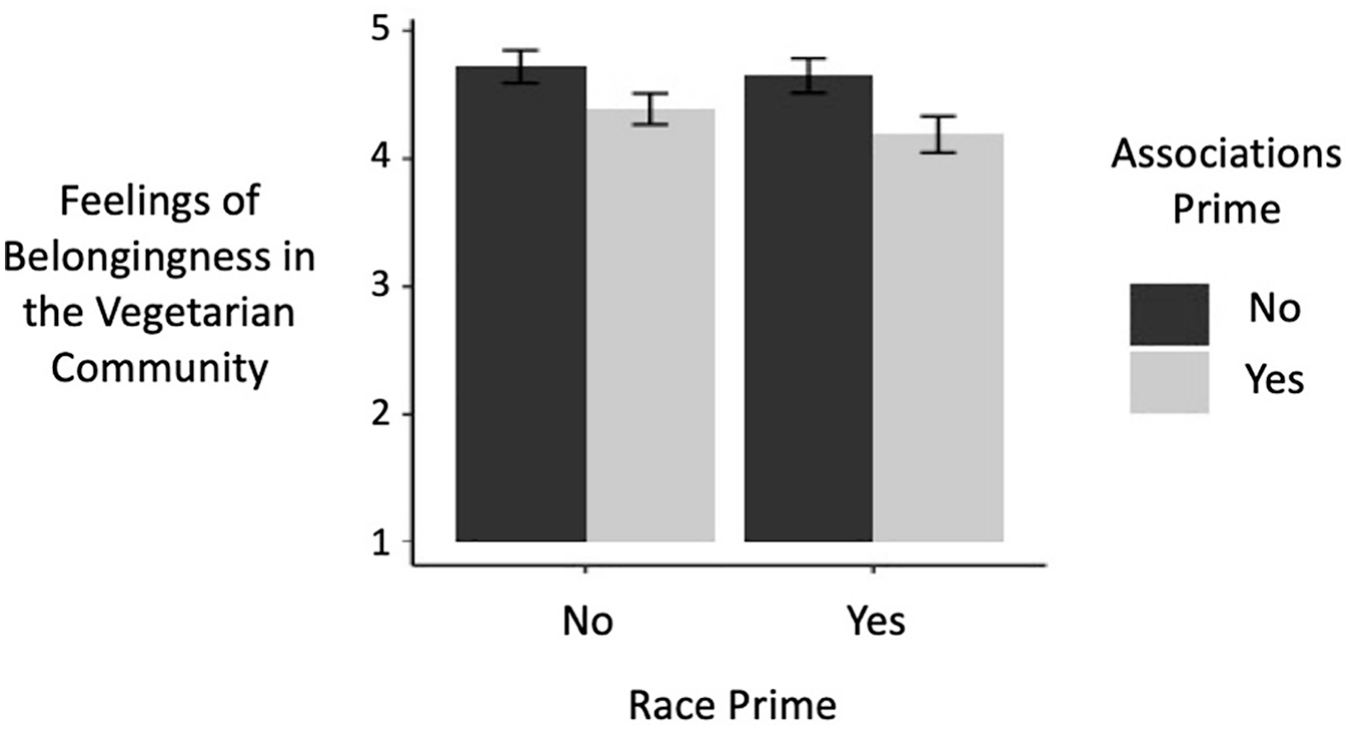
Effects of priming race and race–vegetarianism associations on feelings of belongingness in the vegetarian community in a sample of Black participants in Study 3 (error bars indicate standard errors; belongingness was assessed on a Scale of 1–7).

A two-way ANOVA revealed a significant main effect of race-vegetarianism association prime (i.e., completion of IAT + explicit items) on feelings of belongingness in the vegetarian community, *F*(1, 396) = 9.00, *p* = .003, η_p_^2^ = 0.02, such that participants for whom associations were primed (*M* = 4.29, *SD* = 1.31) reported lower belongingness than did participants for whom associations were not primed (*M* = 4.68, *SD* = 1.33), *d* = 0.30 (see Figure 3). The main effect of race prime, *F*(1, 396) = 0.76, *p* = .384, η_p_^2^ = 0.00, and the interaction effect between the two primes, *F*(1, 396) = 0.23, *p* = .633, η_p_^2^ = 0.00, were not significant.

## Study 4

Results of Study 3 showcase the potential for racial stereotypes about vegetarianism to undermine belongingness among Black individuals. Reflecting on their racial stereotypes about vegetarianism did not influence Black individuals’ interest in becoming a vegetarian but did cause them to feel less belongingness in the vegetarian community. These findings highlight a challenge for racial inclusion.

In Study 4, we pursued a potential solution to increase belongingness. Specifically, through an informative pro-vegetarian slideshow, we tested whether framing vegetarianism as racially inclusive could make it more appealing to Black individuals. As people of color tend to be attuned to potential signs of underrepresentation (Emerson & Murphy, 2014; Purdie-Vaughns et al., 2008), we varied whether or not the slideshow depicted images of Black people. In doing so, we manipulated what situational cues were present to participants, which have been demonstrated to influence behavioral intentions and feelings of belongingness in other stereotyped domains (e.g., Murphy et al., 2007).

We compared the effects of an inclusive frame (i.e., exposure to depictions of both Black and White people) to the effects of a stereotypical frame of vegetarianism (i.e., exposure to depictions of only White people) and, for exploratory descriptive purposes, a neutral control (i.e., no exposure to any frame). As in Study 3, we considered interest and belongingness as two outcomes of interest. We hypothesized that depictions of both Black and White people would cause participants to report greater interest in becoming a vegetarian and greater feelings of belongingness in the vegetarian community than would depictions of only White people.

### Method

This study’s sample size, materials, conditions, exclusion criteria, hypotheses, and analyses were preregistered at https://osf.io/3fnvg.

Data, analysis scripts, and codebook are available at https://osf.io/k67fg/.

#### Participants

In determining this study’s sample size, we accounted for small–medium differences between conditions. A power analysis using G*Power 3.1 specifying an effect of *d* = 0.35 revealed that a total sample of 390 participants would provide 80% power at α = .05, two-tailed. To increase power further, we recruited a generous sample of 600 Black nonvegetarian adults from the United States, recruited via Prolific, from May 20–27, 2021. Participants were prescreened by race and dietary status automatically through Prolific (without participants’ awareness) and reported their race and diet at the end of the survey; we continued data collection until our sample included 600 participants who self-identified as Black and nonvegetarian. After excluding eight participants who failed an attention check in the survey, 592 participants (248 men, 338 women, 6 other) between the ages of 18 and 76 (*M*_age_ = 33.55, *SD* = 11.33) were retained for analyses. This sample provided 80% power to detect small–medium condition effects of *d* = 0.28 at α = .05, two-tailed.

#### Materials

##### Framing of vegetarianism

**Figure 4.**
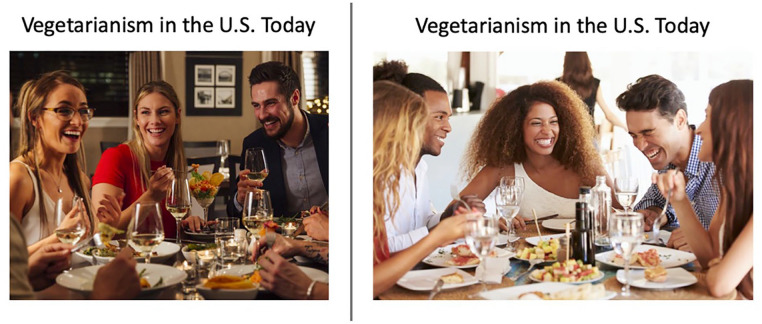
An example slide from each slideshow condition in Study 4 (slide with White people only on the left; slide with both Black and White people on the right).

Through two versions of a slideshow, we manipulated whether a message endorsing vegetarianism depicted (a) images of White people exclusively or (b) images of both Black and White people. Each slideshow included 11 slides focused on the health and environmental benefits of vegetarianism as well as vegetarianism’s rising popularity in the United States. Figure 4 presents an example slide from each slideshow. The full slideshow for each condition is available at https://osf.io/6cxw8/.

##### Measures

Interest in becoming a vegetarian (α = .95) and feelings of belongingness in the vegetarian community (α = .93) were assessed as in Study 3.

#### Procedure

After consenting to take part in this study, participants were randomly assigned to complete one of the three study conditions: (a) a stereotypical condition, in which participants viewed the slideshow of White people before completing outcome measures (interest and belongingness); (b) an inclusive condition, in which participants viewed the slideshow of both Black and White people before completing outcome measures; and (c) a control condition, in which participants completed outcome measures at the start of the study, not having viewed any slideshow. At the end of the survey, participants completed demographic questions.

### Results and Discussion

Interest and belongingness were correlated moderately at *r*(582) = .34, *p* < .001.

**Figure 5.**
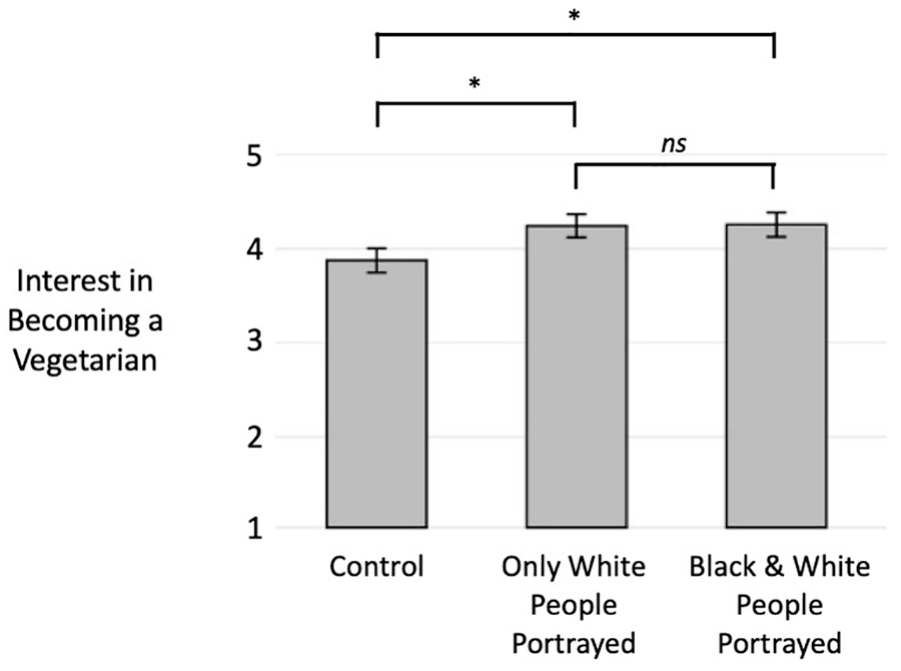
Effects of portraying vegetarianism with White people only versus with both Black and White people on Black participants’ interest in becoming a vegetarian in Study 4 (error bars indicate standard errors; interest was assessed on a scale of 1–7).

For interest, our hypothesis was unsupported (see Figure 5). Pairwise comparisons within a one-way ANOVA revealed no significant difference in interest in becoming a vegetarian between the stereotypical (*M* = 4.24, *SD* = 1.73) and inclusive (*M* = 4.26, *SD* = 1.80) conditions, *t*(589) = 0.07, *p* = .944, 95% CI [−0.34, 0.37], *d* = 0.01, though both conditions were higher in interest than the neutral control (*M* = 3.88, *SD* = 1.81): stereotypical condition *t*(589) = 2.05, *p* = .040, 95% CI [0.02, 0.72], *d* = 0.20, inclusive condition *t*(589) = 2.12, *p* = .034, 95% CI [0.03, 0.73], *d* = 0.21.

**Figure 6.**
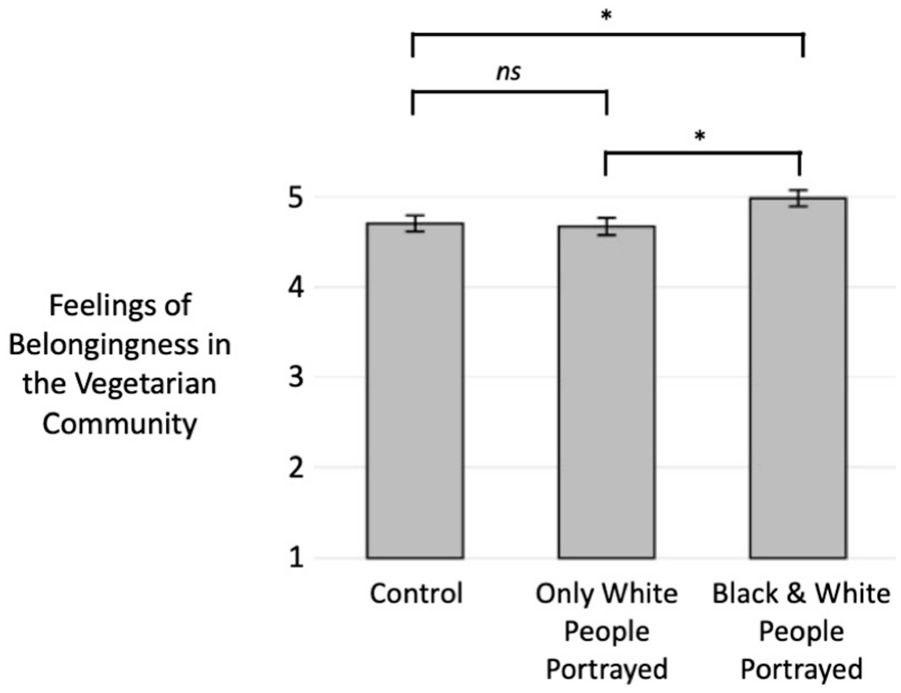
Effects of portraying vegetarianism with White people only versus with both Black and White people on Black participants’ feelings of belongingness in the vegetarian community in Study 4 (error bars indicate standard errors; belongingness was assessed on a scale of 1–7).

For belongingness, meanwhile, our hypothesis was supported (see Figure 6). A pairwise comparison within a one-way ANOVA revealed higher feelings of belongingness in the vegetarian community in the inclusive condition (*M* = 4.99, *SD* = 1.23) than in the stereotypical condition (*M* = 4.67, *SD* = 1.33), *t*(581) = 2.43, *p* = .016, 95% CI [0.06, 0.57], *d* = 0.25. Additional pairwise comparisons revealed higher belongingness in the inclusive condition relative to the neutral control (*M* = 4.71, *SD* = 1.26), *t*(581) = 2.18, *p* = .030, 95% CI [0.03, 0.54], *d* = 0.22, but no difference between the stereotypical condition and the neutral control, *t*(581) = 0.26, *p* = .799, 95% CI [−0.29, 0.22], *d* = 0.03.

## General Discussion

These studies provide three key insights into stereotypes about race and vegetarianism. First, our findings suggest that vegetarianism is markedly racialized. Participants explicitly associated vegetarianism most strongly with White people and least strongly with Black and Latinx people; associations with Asian people were in between these two ends. Strong associations of vegetarianism with Whiteness were observed not only explicitly but also implicitly. Second, experimentally increasing the salience of their race led Black participants to report lower interest in becoming a vegetarian, and increasing the salience of their associations between race and vegetarianism led them to feel less belongingness in the vegetarian community. Third, exposure to racially inclusive messaging about vegetarianism was effective at increasing Black participants’ feelings of belongingness.

This research provides the first quantitative evidence to suggest that people hold strong associations between race and vegetarianism, perceiving vegetarianism as a stereotypically White behavior. Establishing these associations invites new psychological inquiry about stereotypes and eating behavior, which can have meaningful implications for how researchers, advocates, practitioners, and marketers conceptualize and frame vegetarianism to diverse populations. As findings from Study 4 suggest, culturally sensitive efforts to promote more vegetarian consumption among Black people may be valuable for ensuring an inclusive climate. High rates of meat consumption burden chronic disease rates and sustainability goals (Willett et al., 2019). There is great potential for increased consumption of vegetarian and vegetarian-inclined diets to improve human and ecological health, offering one way to support health equity among people of color. Yet racial stereotypes might fuel among Black people a sense that they do not belong in vegetarian spaces. The average person makes over 200 food choices every day, and these decisions unfold across a variety of multifaceted, dynamic, and situationally powerful contexts (Sobal & Bisogni, 2009). Accordingly, even if racial stereotypes have relatively small effects on dietary attitudes, as observed in Studies 3 and 4, such effects may still impart meaningful impacts on health outcomes, sustainability metrics, and feelings of inclusion (or lack thereof) writ large over an entire lifetime of eating. As the role of vegetarianism becomes more pronounced in U.S. culture, among other cultures, a deeper understanding of social psychological processes underlying attitudes toward this diet will be vital to optimize social change and inclusion.

Whereas cues that activate Black people’s own racial identities may lower their interest in becoming a vegetarian, cues that activate stereotypes of vegetarianism with Whiteness may lower Black people’s expectations that they would belong in the vegetarian community. Notably, our data suggest that targeting racial stereotypes about vegetarianism can likely impart more immediate changes in Black individuals’ sense of belongingness in vegetarianism, rather than in their interest in adopting a vegetarian diet. We found consistent evidence for this point across Studies 3 and 4, as belongingness decreased (but interest remained constant) in response to a race-vegetarianism association prime relative to control in Study 3, whereas belongingness increased (but interest remained constant) in response to a racially inclusive frame relative to a stereotypical frame in Study 4. Together, these studies furthermore highlight that systematic manipulations can either decrease *or* increase how much belongingness Black people feel in the vegetarian community. That racially inclusive messaging increased Black individuals’ feelings of belongingness in vegetarianism aligns with other research demonstrating analogous effects for women in math, science, and engineering settings (Murphy et al., 2007). Moreover, it supports existing evidence suggesting that social identity threat diminishes among people of color when they perceive a community as including a significant number of in-group members, or “critical mass” (Emerson & Murphy, 2014). Over 80% of people who become vegetarian eventually lapse back to eating meat (Herzog, 2014), and evidence suggests that lacking a sense of belongingness in a new community increases minority group members’ chances of withdrawing from the community (Murphy et al., 2020). Interventions aimed at improving belongingness for new vegetarians of color might yield novel ways of supporting lasting behavior change.

Our findings may also be situated within identity-based motivation perspectives positing that stereotypes about group norms may deter people from engaging in behaviors linked to an out-group, even when such behaviors may improve one’s well-being (e.g., Oyserman, 2009; Oyserman et al., 2007). In light of identity-based perspectives and other findings on social identity threat (e.g., Murphy et al., 2007), it is sensible to observe that race primes reduced Black individuals’ interest in becoming a vegetarian but it is less clear why neither priming nor reframing racial stereotypes about vegetarianism influenced interest. Of note, the two framing conditions in Study 4 differed significantly from the control. Given that the control condition likely reflected participants’ default associations—which commonly link vegetarianism to Whiteness, as suggested by Studies 1–3—the two priming conditions, while varying in racial representation, may have made social and/or gendered aspects of vegetarianism salient. That is, across priming conditions, the images overwhelmingly depicted individuals interacting with others and included relatively gender-balanced images (e.g., inclusive of men, women, boys, and girls). Thus, relative to the control, which reflected participants’ default associations, both priming conditions appeared to have benefited interest; both priming conditions may have expanded associations with vegetarianism in ways that are likely to be culturally valued by Black people. For example, making social aspects of vegetarianism salient may have also made interdependence and collectivism salient, which are especially valued and self-relevant for Black people (see Brannon et al., 2015). Further research investigating experimental interventions tied to emphasizing interdependent or collectivistic aspects of vegetarianism for fostering interest in behavior change would be valuable.

Another avenue for future research may be to further manipulate social identity contingencies (Steele et al., 2002) in vegetarianism by varying features of the immediate social context surrounding participants (e.g., racial composition of a specific setting), the language with which vegetarianism is framed, or the cuisine type depicted visually in vegetarian messages. Indeed, research that has examined belonging among women and people of color in mainstream settings (e.g., male-dominated disciplines, schools, and workplaces) has revealed positive consequences tied to inclusive social contextual factors (e.g., in-group representations, value-diversity ideologies, and engagement with multicultural practices, Brannon & Lin, 2021; Murphy et al., 2007; Purdie-Vaughns et al., 2008), and similar effects may occur within the domain of diet. Such efforts may be complemented by framing vegetarianism as historically has been central to African American culture prior to the European Slave Trade (e.g., Crimarco et al., 2020). Food traditions in Black cultures often have stemmed from experiencing and overcoming oppression, and emphasizing that plant-based diets have roots in traditional African American cultures—and that consuming these diets gives individuals agency in enabling health improvements—may be promising communication strategies.

Gender presents an identity domain with high capacity to intersect with racial stereotypes about vegetarian. Meat is associated with masculinity whereas vegetarianism is tied to femininity (Rothgerber, 2013). Race, too, is gendered, with Black individuals, in particular, being perceived as more masculine than are members of other groups (Johnson et al., 2012). Thus, it is possible that the content of gendered and racial stereotypes about vegetarianism overlap and may influence people’s feelings and behaviors in tandem. Further research on vegetarianism can benefit from evaluating gender alongside race.

Understanding these basic phenomena can inform optimal strategies for developing intervention efforts and materials that encourage more healthful and environmentally sustainable eating patterns. Through consumption behaviors such as purchasing and eating food, individuals enact, affirm, and construct identities (Arsel & Thompson, 2010; Reed et al., 2012). Importantly, consumption behaviors are often social in their capacity to stimulate shared social connections and experiences as well as social support and belongingness (Arsel & Thompson, 2010). Thus, the way in which people of color construe vegetarianism as a stereotypically White behavior may intersect with their own consumption-based identity and their deeper social identities in which consumption is embedded. To cultivate a more inclusive atmosphere that minimizes feelings of racial group threat or self-inconsistency, researchers and interventionists may benefit from considering identity-based consumer behavior frameworks (e.g., Arsel & Thompson, 2010; Oyserman, 2009; Reed et al., 2012) and the perceptual and linguistic cues, both explicit and subtle, that prime components of involved social identity processes.

The ways in which Black people, and other people of color, engage with racial and dietary identities that may feel incompatible are in great need of future research. Investigating this matter specifically among individuals who are already vegetarian may be particularly interesting in light of dual identity and acculturation perspectives (e.g., Baysu et al., 2011; Berry, 2005; Ryder et al., 2000). For example, even if Black people come to feel included in vegetarianism, their social identity as not just a vegetarian but more intersectionally as a *Black* vegetarian (i.e., a dual identity) may be salient and heighten their alertness to within-group power inequalities (Dovidio et al., 2016). Such inquiry is opportune, as—despite stereotypes suggesting the reverse—Black people appear to be embracing vegetarian eating patterns at *higher* rates than are other racial groups (“Why Black Americans are More Likely to be Vegan,” 2020).

Notably, nevertheless, there are clear systemic factors that may disproportionately undermine the ability to adopt and/or maintain a vegetarian diet among people of color compared with White people. Systemic racial inequalities in access to food undermine choice among Black individuals (Walker et al., 2010), yielding for them less access to financial resources, insufficient food options in their proximity, and less available meal-time preparation. Moreover, greater experiences of stress from racial discrimination among people of color may contribute to emotional eating (Longmire-Avital & McQueen, 2019); high access to unhealthful food and a lack of access to healthful food may interact with discrimination among people of color to explain links between race and eating behavior. Efforts to reduce racial disparities in health through the promotion of vegetarian eating accordingly can only be optimized by not only combating racialized perceptions of vegetarianism but also addressing structural and social factors that directly influence eating behaviors.

### Limitations

One limitation of this research is that, in Studies 3 and 4, interest in becoming a vegetarian was operationalized via self-report, rather than a documentation of behavior change over time. Thus, the current findings may only speak to individuals’ *intentions* to become vegetarian; additional data from longitudinal research tracking eating behavior over time would be valuable. A second limitation is that, as Studies 3 and 4 included only Black participants, it remains unknown whether similar effects would occur for other people of color, such as Latinx or Asian individuals. Future research should examine the effects of racial/ethnic stereotypes about vegetarianism among a wider variety of racial and ethnic groups to test the generalizability of basic processes. A limitation specific to Study 3 is that completion of the racial centrality scale, as part of the race prime, could have undermined the strength of this prime among participants with low centrality. A limitation specific to Study 4 is the possibility of demand characteristics, as participants may have become aware of this study’s aims once they arrived at the intentions and belongingness scales. A second limitation for Study 4 is that we did not pretest the two slideshows used. While we aimed for the two slideshows to be matched in terms of core factors (e.g., age, gender, and affect of people depicted), the slideshows may have differed meaningfully along influential dimensions, such as perceptions of attractiveness, family orientation, or racial prototypicality. A third limitation of Study 4 is that the people depicted in the slideshows tended to show signs of being of higher social class, which could have reinforced stereotypes about class and vegetarianism. More broadly, our studies are limited in having not accounted empirically for intersections of race and class; further work on vegetarianism would benefit from disentangling stereotypes surrounding race and class from one another.

While our use of both MTurk and Prolific for participant recruitment helped confirm replicability in race–vegetarianism associations across online populations, uncertainties remain about the generalizability of our findings to participants from distinct subcultures across the United States who may be less likely to participate in online survey platforms. An additional, clear limitation to these studies’ generalizability is that all participants resided in the United States, so these findings may not be generalizable across countries and cultures.

## Conclusion

Stereotypes about race and vegetarianism offer a rich area for social psychological inquiry. U.S. adults associate vegetarianism with Whiteness very strongly, both explicitly and implicitly, and these associations may make vegetarianism feel noninclusive to people of color. Framing vegetarianism as a community that people of any race can join offers a promising strategy for overcoming existing stereotypes and fostering a sense of belongingness.

Much evidence suggests that vegetarian diets improve human health and promote environmental sustainability (Willett et al., 2019), and vegetarian dietary behaviors appear to be becoming increasingly mainstream across Western cultures. By understanding the intersections of racial stereotypes and eating behavior, we become more empowered to cultivate a maximally inclusive food environment.

## Supplemental Material

sj-docx-1-psp-10.1177_01461672221099392 – Supplemental material for Racialized Perceptions of Vegetarianism: Stereotypical Associations That Undermine Inclusion in Eating Behaviors Click here for additional data file. Supplemental material, sj-docx-1-psp-10.1177_01461672221099392 for Racialized Perceptions of Vegetarianism: Stereotypical Associations That Undermine Inclusion in Eating Behaviors by Daniel L. Rosenfeld, Tiffany N. Brannon and A. Janet Tomiyama in Personality and Social Psychology Bulletin

## Notes

1. Although our focus is on psychosocial factors, important to note is that structural factors influence eating behavior as well. For example, some racial groups are more concentrated in neighborhoods with less accessibility to sufficient food options. We emphasize that racial stereotypes about vegetarianism could be influential *in addition to* structural factors, not instead of them.
