# Supplementary material for: Racialized Perceptions of Vegetarianism: Stereotypical Associations That Undermine Inclusion in Eating Behaviors
Source: Pers Soc Psychol Bull. 2022 Jul 7;49(11):1601–14. doi: 10.1177/01461672221099392 (PMC10517590; doi:10.1177/01461672221099392)
Supplement: sj-docx-1-psp-10.1177_01461672221099392 – Supplemental material for Racialized Perceptions of Vegetarianism: Stereotypical Associations That Undermine Inclusion in Eating Behaviors [file sj-docx-1-psp-10.1177_01461672221099392.docx]

**Study 1**

Please select your gender:

- Man
- Woman
- Non-Binary
- Other

Please bring to mind people who eat a vegetarian diet. In your mind, how “vegetarian” are people who belong to the following racial groups? That is, how strongly are they identified with vegetarianism and all things vegetarian?

|  | Not At All | Very Little | Somewhat | Quite A Bit | Very Much |
| --- | --- | --- | --- | --- | --- |
| White people |  |  |  |  |  |
| Black people |  |  |  |  |  |
| Latino/a people |  |  |  |  |  |
| Asian people |  |  |  |  |  |

When you think of a vegetarian, how much do you think of someone who is...

|  | Not At All | Very Little | Somewhat | Quite A Bit | Very Much |
| --- | --- | --- | --- | --- | --- |
| White |  |  |  |  |  |
| Black |  |  |  |  |  |
| Latino/a |  |  |  |  |  |
| Asian |  |  |  |  |  |

Please specify your age, in years, below:

________________________________________________________________

**Study 2**

Please specify your age, in years, below:

________________________________________________________________

Please select your gender:

- Man
- Woman
- Non-Binary
- Other

Which race/ethnicity best describes you?

- White / Caucasian
- Black / African American
- Hispanic or Latinx
- Asian or Pacific Islander
- Native American
- Other

Are you a vegetarian?

- Yes
- No

Please bring to mind people who eat a vegetarian diet. In your mind, how “vegetarian” are people who belong to the following racial groups? That is, how strongly are they identified with vegetarianism and all things vegetarian?

|  | Not At All | Very Little | Somewhat | Quite A Bit | Very Much |
| --- | --- | --- | --- | --- | --- |
| White people |  |  |  |  |  |
| Black people |  |  |  |  |  |

When you think of a vegetarian, how much do you think of someone who is...

|  | Not At All | Very Little | Somewhat | Quite A Bit | Very Much |
| --- | --- | --- | --- | --- | --- |
| White |  |  |  |  |  |
| Black |  |  |  |  |  |

Implicit association test is available for download at <https://osf.io/782nh/>

**Study 3**

Which race/ethnicity best describes you?

- White
- Black
- Hispanic / Latinx
- Asian / Pacific Islander
- Other

Please take a moment to reflect on what you think it means to be a Black person. Reflect on what role race plays in your life. When you are ready, please proceed with the survey.

Please indicate how strongly you agree or disagree with each of the following statements.

|  | Strongly Disagree | Disagree | Somewhat Disagree | Neither Agree nor Disagree | Somewhat Agree | Agree | Strongly Agree |
| --- | --- | --- | --- | --- | --- | --- | --- |
| In general, being Black is an important part of my self-image. |  |  |  |  |  |  |  |
| I feel a strong sense of belonging to the Black community. |  |  |  |  |  |  |  |
| I have a strong attachment to other Black people. |  |  |  |  |  |  |  |
| Being Black is an important reflection of who I am. |  |  |  |  |  |  |  |

Please bring to mind people who eat a vegetarian diet. In your mind, how “vegetarian” are people who belong to the following racial groups? That is, how strongly are they identified with vegetarianism and all things vegetarian?

|  | Not At All | Very Little | Somewhat | Quite A Bit | Very Much |
| --- | --- | --- | --- | --- | --- |
| White people |  |  |  |  |  |
| Black people |  |  |  |  |  |

When you think of a vegetarian, how much do you think of someone who is...

|  | Not At All | Very Little | Somewhat | Quite A Bit | Very Much |
| --- | --- | --- | --- | --- | --- |
| White |  |  |  |  |  |
| Black |  |  |  |  |  |

Implicit association test is available for download at <https://osf.io/782nh/>

Are you a vegetarian?

- Yes
- No

Please indicate how strongly you agree or disagree with each of the following statements.

|  | Strongly Disagree | Disagree | Somewhat Disagree | Neither Agree nor Disagree | Somewhat Agree | Agree | Strongly Agree |
| --- | --- | --- | --- | --- | --- | --- | --- |
| I am open to becoming a vegetarian. |  |  |  |  |  |  |  |
| I can imagine myself becoming a vegetarian someday. |  |  |  |  |  |  |  |
| I would like to try out a vegetarian diet. |  |  |  |  |  |  |  |
| I want to become a vegetarian in the near future. |  |  |  |  |  |  |  |

If I were to become a vegetarian...

|  | Strongly Disagree | Disagree | Somewhat Disagree | Neither Agree nor Disagree | Somewhat Agree | Agree | Strongly Agree |
| --- | --- | --- | --- | --- | --- | --- | --- |
| I would feel included by other vegetarians. |  |  |  |  |  |  |  |
| I would feel a sense of belonging in the vegetarian community. |  |  |  |  |  |  |  |
| I would feel close bonds with other vegetarians. |  |  |  |  |  |  |  |
| I would feel accepted by other vegetarians. |  |  |  |  |  |  |  |
| I would feel connected with other vegetarians. |  |  |  |  |  |  |  |
| I would feel like an outsider from other vegetarians. |  |  |  |  |  |  |  |
| I would feel distant from other vegetarians. |  |  |  |  |  |  |  |
| I would feel like a stranger around other vegetarians. |  |  |  |  |  |  |  |

Please specify your age, in years, below:

________________________________________________________________

Please select your gender:

- Man
- Woman
- Non-Binary
- Other

**Study 4**

The full slideshow for each condition is available at <https://osf.io/6cxw8/>

Are you a vegetarian?

- Yes
- No

Please indicate how strongly you agree or disagree with each of the following statements.

|  | Strongly Disagree | Disagree | Somewhat Disagree | Neither Agree nor Disagree | Somewhat Agree | Agree | Strongly Agree |
| --- | --- | --- | --- | --- | --- | --- | --- |
| I am open to becoming a vegetarian. |  |  |  |  |  |  |  |
| I can imagine myself becoming a vegetarian someday. |  |  |  |  |  |  |  |
| I would like to try out a vegetarian diet. |  |  |  |  |  |  |  |
| I want to become a vegetarian in the near future. |  |  |  |  |  |  |  |

If I were to become a vegetarian...

|  | Strongly Disagree | Disagree | Somewhat Disagree | Neither Agree nor Disagree | Somewhat Agree | Agree | Strongly Agree |
| --- | --- | --- | --- | --- | --- | --- | --- |
| I would feel included by other vegetarians. |  |  |  |  |  |  |  |
| I would feel a sense of belonging in the vegetarian community. |  |  |  |  |  |  |  |
| I would feel close bonds with other vegetarians. |  |  |  |  |  |  |  |
| I would feel accepted by other vegetarians. |  |  |  |  |  |  |  |
| I would feel connected with other vegetarians. |  |  |  |  |  |  |  |
| I would feel like an outsider from other vegetarians. |  |  |  |  |  |  |  |
| I would feel distant from other vegetarians. |  |  |  |  |  |  |  |
| I would feel like a stranger around other vegetarians. |  |  |  |  |  |  |  |

Please specify your age, in years, below:

________________________________________________________________

Please select your gender:

- Man
- Woman
- Non-Binary
- Other

Which race/ethnicity best describes you?

- White
- Black
- Hispanic / Latinx
- Asian / Pacific Islander
- Other
